# Administration of nintedanib after discontinuation for acute exacerbation of idiopathic pulmonary fibrosis: a case report

**DOI:** 10.1186/s12890-016-0201-9

**Published:** 2016-03-03

**Authors:** Satoshi Ikeda, Akimasa Sekine, Tomohisa Baba, Hideaki Yamakawa, Masato Morita, Hideya Kitamura, Takashi Ogura

**Affiliations:** Department of Respiratory Medicine, Kanagawa Cardiovascular and Respiratory Center, Tomioka-Higashi 6-16-1, Kanazawa-ku, Yokohama, 236-0051 Japan

**Keywords:** Idiopathic pulmonary fibrosis, Acute exacerbation, Nintedanib

## Abstract

**Background:**

Nintedanib is a multi-target receptor tyrosine kinase inhibitor. In two recent randomized phase 3 trials (INPULSIS™-1 and -2), it has been shown to slow the disease progression of idiopathic pulmonary fibrosis (IPF) by reducing the decline in the forced vital capacity (FVC). Although the INPULSIS™ trials indicate that nintedanib may serve to prevent acute exacerbations or delay the time to the first acute exacerbation, a certain number of IPF patients develop acute exacerbations while receiving nintedanib. However, there has been no report on the readministration of nintedanib in IPF patients who develop acute exacerbations during initial treatment with nintedanib.

**Case Presentation:**

A 64-year-old man with IPF had nintedanib added to his ongoing pirfenidone therapy. He developed dyspnea after 65 days and presented with hypoxemia after 68 days. At presentation, chest computed tomography showed newly developed diffuse ground glass opacities with the pre-existing subpleural reticular shadows. Because of the absence of infection or other potential causative factors, we diagnosed an acute exacerbation of IPF. Nintedanib was temporarily discontinued and the acute exacerbation was successfully managed with intensive treatment. We re-initiated nintedanib 30 days after cessation, which helped stabilize his FVC for 8 months. Nintedanib was safely continued for 28 months until he died of a bacterial infection.

**Conclusion:**

To the best of our our knowledge, this is the first reported case of an acute exacerbation of IPF during nintedanib treatment, wherein nintedanib was safely and successfully restarted after treatment of the acute exacerbation. Our case indicates that nintedanib can be safely resumed and a desired effect on FVC can be obtained, even in IPF patients who develop acute exacerbations. However, we recommend close monitoring and appropriate measures until the long-term safety profile is clarified.

## Background

Nintedanib is a multi-target receptor tyrosine kinase inhibitor that inhibits vascular endothelial growth factor (VEGF), platelet-derived growth factor (PDGF), and fibroblast growth factor (FGF) [1]. In patients with idiopathic pulmonary fibrosis (IPF), two replicate randomized phase 3 trials, INPULSIS™-1 and -2, demonstrated that nintedanib reduced the decline in the forced vital capacity (FVC), with a manageable side-effect profile [2]. Furthermore, the results of an earlier phase 2 trial suggested that nintedanib was associated with fewer acute exacerbations of IPF [3]. In the INPULSIS™-2 trial, there was a significant increase in time to the first acute exacerbation in the nintedanib group when compared with that in the placebo group; however, this was not observed in the INPULSIS™-1 trial. Although a prespecified verification by a central adjudication committee showed that the risk of acute exacerbation of IPF was significantly lower in the nintedanib group than in the placebo group [4], a certain number of IPF patients developed acute exacerbation in the nintedanib group. However, there have been no reports on the readministration of nintedanib in patients who developed acute exacerbation during initial treatment with nintedanib.

## Case Presentation

### Case report

**Fig. 1:**
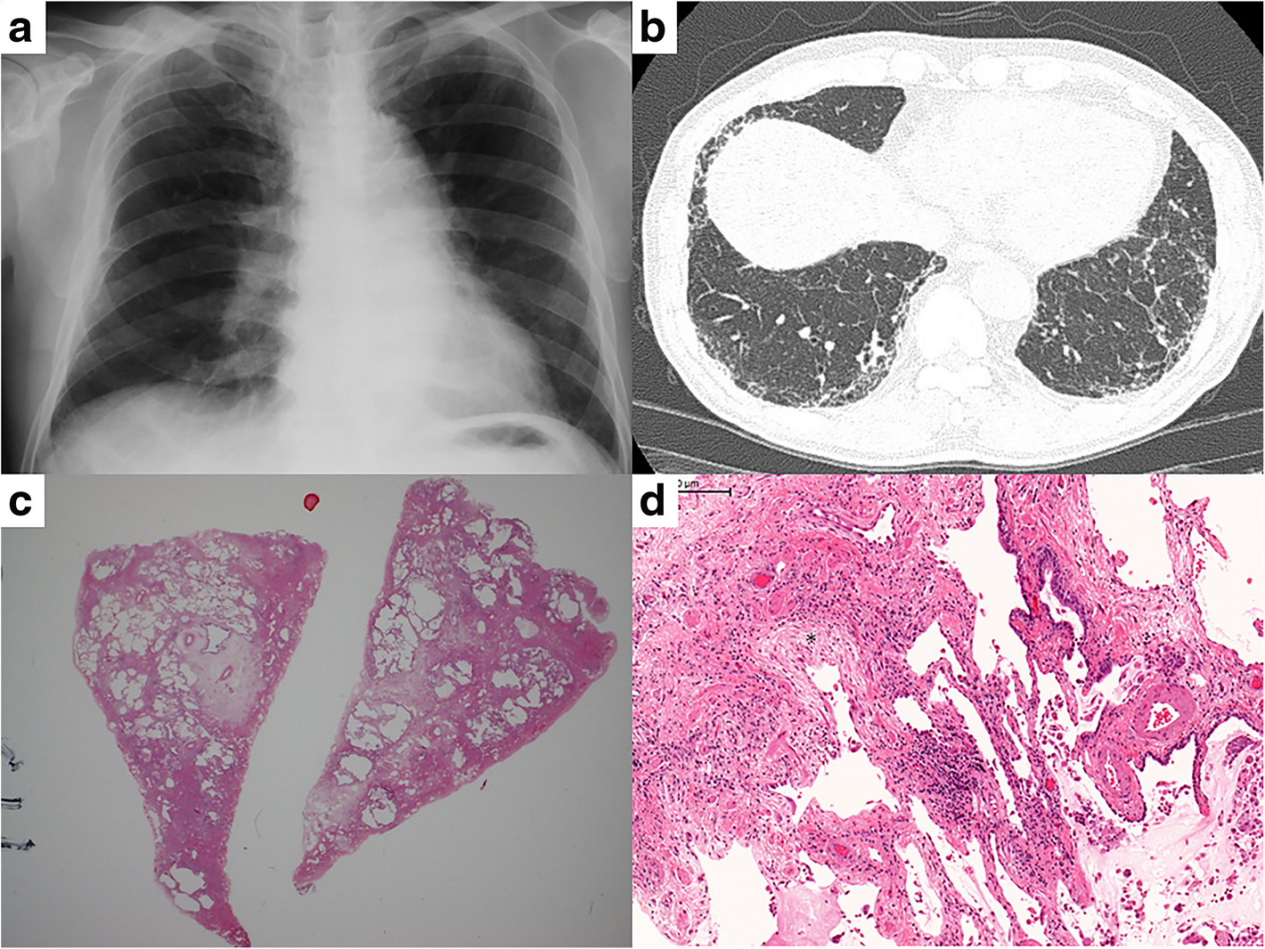
Representative photographs from radiographic and microscopic examination before starting nintedanib. Chest X-ray (**a**) and chest high resolution computed tomography (**b**) before starting nintedanib revealed subpleural reticular shadowing that was predominantly in the lower lobes, without honeycombing. Pathological examination of a surgical lung biopsy specimen showed subpleural fibrosis and microscopic honeycombing (**c**; hematoxylin and eosin stain, low-power field) with patchy distribution of dense fibrosis, fibroblastic foci (*), and normal lung (**d**; hematoxylin and eosin stain, high-power field). Thus, the diagnosis of idiopathic pulmonary fibrosis was confirmed

We present the case of a 64-year-old male former smoker with IPF. He presented to our hospital complaining of dry cough in September 2005. Chest computed tomography (CT) at that time revealed subpleural reticular shadow predominantly in the lower lobes, without apparent honeycombing (Fig. 1a, b). Surgical lung biopsy showed patchy distribution of dense fibrosis, fibroblastic foci, and normal lung with microscopic honeycombing (Fig. 1c, d). Thus, the diagnosis of IPF was confirmed. Although pirfenidone was administered as the first-line treatment, his FVC gradually decreased. Therefore, in October 2010, we added nintedanib to the ongoing pirfenidone therapy. Nintedanib was administered at a dose of 50 mg twice daily for 28 days as part of a phase 2 dose escalation trial [5].

**Fig. 2:**
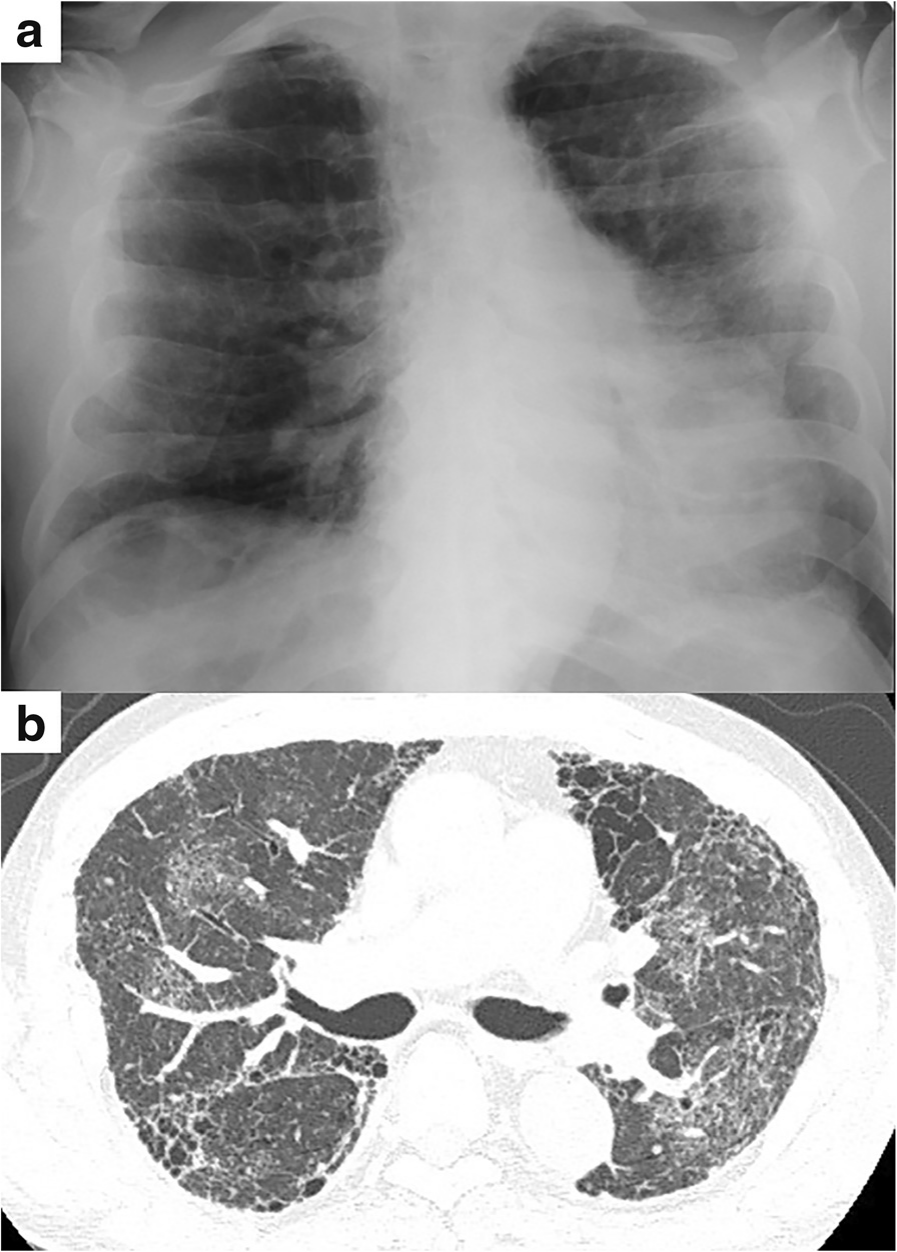
Chest X-ray and high resolution computed tomography at acute exacerbation of idiopathic pulmonary fibrosis onset. Chest X-ray (**a**) and high resolution computed tomography (**b**) at the onset of acute exacerbation. On day 68, chest high resolution computed tomography showed newly developed, diffuse, ground glass opacities

From October 2011, nintedanib was initiated and continuously administered at a dose of 150 mg twice daily. He developed dyspnea 65 days after initiating the treatment dose of 150 mg twice daily and presented to our hospital on day 68. Chest CT showed newly developed diffuse ground glass opacities (Fig. 2). He had hypoxemia with a resting partial pressure of oxygen in arterial blood of 53.6 torr on room air. Laboratory investigation revealed high serum levels of Krebs von den Lungen-6 (1114 U/mL), surfactant protein D (269.5 ng/mL), and lactate dehydrogenase (258 IU/L). The possibility of infection was eliminated based on the results of sputum and blood culture, serum β-D glucan, and cytomegalovirus antigen testing. The possibilities of left-sided heart failure and pulmonary embolism were also ruled out, thus we diagnosed an acute exacerbation of IPF. In accordance with the study protocol, nintedanib was immediately discontinued and intensive treatment comprising methyl prednisolone pulse therapy, direct hemoperfusion using a polymyxin B immobilized fiber column, intravenous cyclophosphamide, and cyclosporine was initiated. Subsequently, his clinical symptoms, PaO_2_/FiO_2_ ratio, and imaging findings gradually improved.

**Fig. 3:**
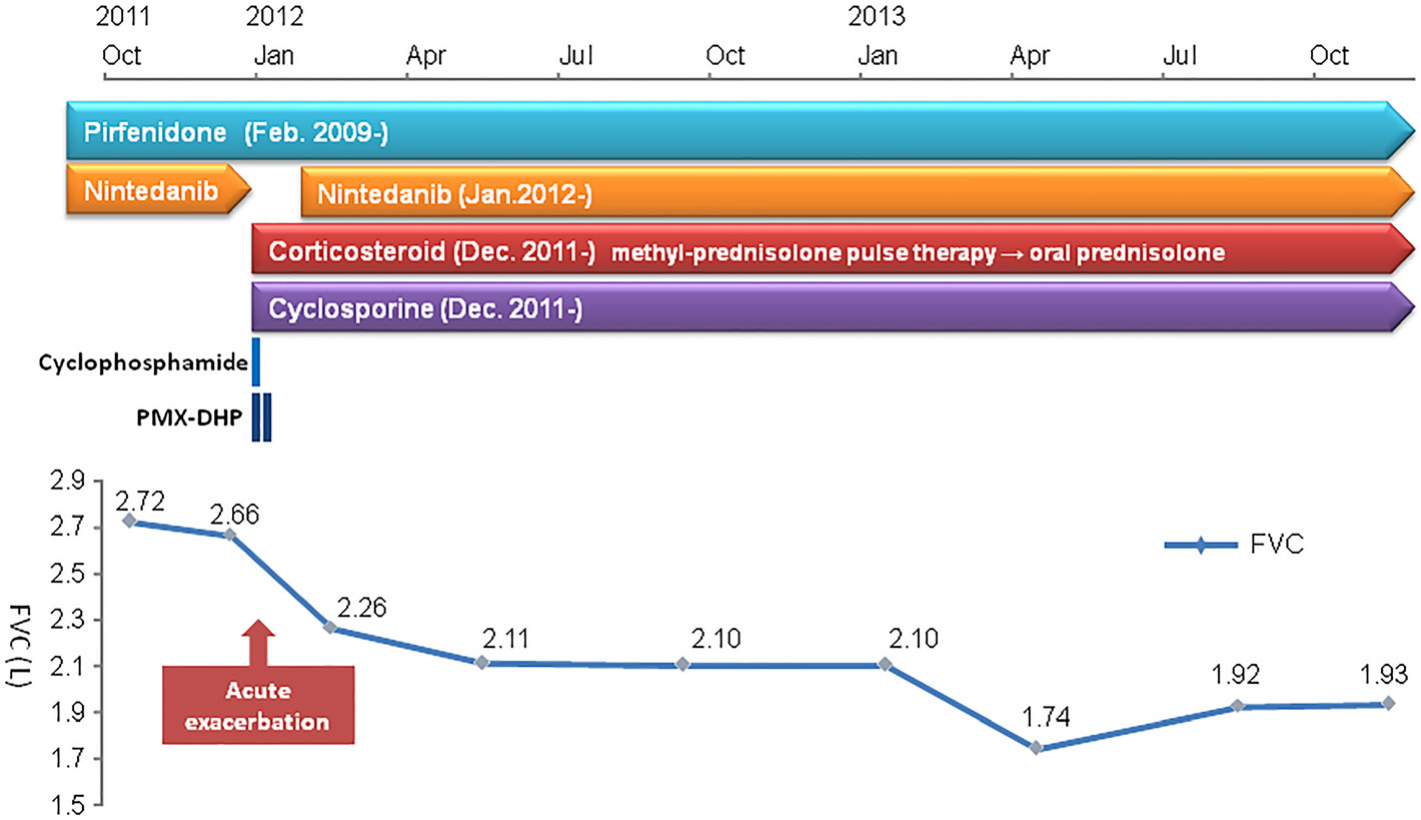
Chronological change in forced vital capacity before and after acute exacerbation of idiopathic pulmonary fibrosis. Under careful observation, nintedanib was restarted 30 days after cessation. Although the forced vital capacity was temporally decreased after the acute exacerbation, it remained stable from May 2012 to January 2013. Abbreviations; PMX-DHP, direct hemoperfusion using a polymyxin B immobilized fiber column

Under careful observation, nintedanib was readministered at a dose of 150 mg twice daily, 30 days after it had been stopped. Although his FVC temporarily decreased after the acute exacerbation, it remained stable from May 2012 to January 2013 (2.11 L to 2.10 L) (Fig. 3). Nintedanib was safely continued without the recurrence of acute exacerbations until he died of a bacterial infection in February 2014.

## Discussion

To the best of our knowledge, this is the first reported case of nintedanib being successfully and safely readministered for IPF after being stopped because of an acute exacerbation during initial treatment. Although it remains to be determined whether nintedanib can induce acute exacerbation of IPF, we temporarily discontinued the treatment because of this possibility and were able to resume nintedanib without the recurrence of acute exacerbation. The clinical course of the present case indicates that nintedanib itself does not induce acute exacerbation of IPF.

Some previous reports support the supposition that nintedanib may not induce acute exacerbation of IPF. First, although it is unknown whether the inhibition of PDGF and FGF receptors can induce exacerbation of interstitial lung disease, studies have indicated that bevacizumab, a recombinant humanized monoclonal antibody against VEGF, does not increase the risk of such exacerbations [6, 7]. Second, in the phase 3 LUME-Lung trials, which assessed the efficacy and safety of nintedanib in addition to docetaxel (LUME-Lung-1) [8] or pemetrexed (LUME-Lung-2) [9] as the second-line therapy for non-small-cell lung cancer, the occurrence of drug-induced interstitial lung disease did not differ between the nintedanib and placebo groups (LUME-Lung-1: 1.4 and 0.8 %, respectively; LUME-Lung-2: 0.6 and 0.8 %, respectively). Although these are only the preliminary results of ongoing clinical studies, they indicate that nintedanib itself does not cause interstitial lung disease.

Acute exacerbations of IPF are generally severe and life threatening, with a reported median survival of only 2.2 months after their onset [10]. To date, it remains unclear whether nintedanib offers the desired beneficial effect in such patients. However, in the present case, there was an apparent inhibitory effect on FVC reduction over an 8-month period after the readministration of nintedanib. A report on the interim analysis of nintedanib in an open-label extension of the INPULSIS™ trials (INPULSIS™-ON) [11] revealed that it provided a positive effect on slowing disease progression for 2 years. Together with the results of the present case, we believe that it is reasonable to assume that the readministration of nintedanib may slow disease progression, even in patients with IPF and a history of acute exacerbation during therapy.

## Conclusions

Our case indicates that nintedanib can be safely resumed and expected to achieve the desirable clinical effect on FVC in patients with IPF who develop acute exacerbation during treatment. However, close monitoring will be needed until its long-term safety profile is elucidated.

### Consent

Written informed consent was obtained from the eldest son of the patient for publication of this case report and any accompanying images. A copy of the written consent is available for review by the Editor of this journal.

## Abbreviations

AE: acute exacerbation
CT: computed tomography
FGF: fibroblast growth factor
FVC: forced vital capacity
ILD: interstitial lung disease
IPF: idiopathic pulmonary fibrosis
PDGF: platelet-derived growth factor
VEGF: vascular endothelial growth factor
